# Enhanced Modulation Bandwidth by Delayed Push–Pull Modulated DFB Lasers

**DOI:** 10.3390/mi14030633

**Published:** 2023-03-10

**Authors:** Jiewen Chi, Xun Li, Chuanning Niu, Jia Zhao

**Affiliations:** 1 School of Information Science and Engineering, Shandong University, Qingdao 266237, China; 2 Department of Electrical and Computer Engineering, McMaster University, Hamilton, ON L8S 4K1, Canada

**Keywords:** DFB, DML, PPM, DPPM, CPR, PPR

## Abstract

The bandwidth of a distributed feedback (DFB) directly modulated laser (DML) is limited by its carrier–photon resonance (CPR) frequency. A viable approach to break the bottleneck is to introduce a photon–photon resonance (PPR), since the PPR can happen at a much higher frequency than the CPR. Among the many structures that can possibly generate the PPR, the dual-sectional push–pull modulated (PPM) DFB is of particular interest for its fabrication cost-effectiveness as no regrowth is required. The PPR in the PPM DFB, however, usually shows a rapid roll-off on both edges, which brings in an indentation on the lower frequency side of the PPR peak and, consequently, cuts off the bandwidth. To compensate for this dip, we introduce a detuned PPR and restart the CPR response by exploiting a time delay between the differential signals applied to the PPM DFB. Our simulation result shows that the broadened PPR peak and the restarted CPR response indeed mitigate the dip and effectively expand the PPM-DFB’s bandwidth to approximately 50 GHz, a value double that of the conventional (single-sectional) DFB DML.

## 1. Introduction

Cost-effective light sources are highly demanded for the broad deployment of high-speed datalinks, telecommunication access networks and wireless communication systems in their front- and middle-haul links [1,2,3,4,5]. Directly modulated lasers (DMLs), as opposed to the externally modulated lasers (EMLs) [6,7,8,9,10], are preferred for their low fabrication cost and high yield since there are no complicated monolithic integration technologies, such as butt-joint regrowth or selective area growth, involved [11,12]. To accommodate the high-speed modulation requirement in the aforementioned applications, a significant amount of effort has been devoted to extending the modulation bandwidth of the distributed feedback (DFB) DMLs mainly by raising their relaxation oscillation frequency caused by the carrier–photon resonance (CPR) [13,14,15]. The single-sectional DFB DML seems to have reached the upper limit of its modulation bandwidth (~25 GHz), and most of the recent works are focused on various multiple-sectional DFB-DML designs that exploit the photon–photon resonance (PPR) to further enlarge the bandwidth [16,17,18,19,20,21,22,23]. Among those experimentally demonstrated and/or theoretically proposed multiple-sectional DFB structures [11,18,24,25,26,27,28,29,30,31,32], the dual-sectional push–pull modulated (PPM) DFB is particularly attractive [33] because it does not need any regrowth due to the uniform active region design in both sections. All other multiple-sectional DFB DMLs, however, need the monolithic integration technique, which weakens their competitiveness on cost effectiveness as compared to EMLs.

The PPM DFB can potentially extend the modulation bandwidth since its PPR can appear at a much higher frequency than the CPR [34,35]. However, the modulation response of the PPM DFB has a huge indentation on the lower frequency side of the PPR peak [36], which makes the PPR ineffective as the bandwidth will still be cut off. A second order Bragg grating, replacing the first order grating in the PPM DFB, alleviates the indentation [37,38]. However, the improvement is not sufficient. Besides, the PPR in the second order grating PPM DFB is usually too high, and it is difficult to raise the normalized coupling coefficient any further, not to mention that its associated radiation loss will inevitably bring in an increased threshold current and a reduced slope efficiency [39,40,41].

In this work, we attempt to detune the PPR and restart the CPR response by introducing a time delay between the two signals applied to the dual-sectional DFB in differential mode. With such a delayed push–pull modulation (DPPM), the PPR of the DFB can be heavily damped due to the detuning, and the CPR response will restart. Hence, the broadened PPR peak and the restarted CPR response can help to fill up the dip. Once a proper delay is introduced for a given PPR frequency, the indentation on the lower frequency side of the PPR peak can be eliminated. A flattened modulation response is, therefore, obtained with a broad bandwidth range determined by the PPR frequency. As a parameter that can be changed freely in operation, the delay time can always be adjusted to achieve the best laser performance under high-speed modulation despite possible variations of laser structural and/or material parameters in fabrication. Therefore, the yield is less of a concern through this approach. In this sense, the extra effort paid to introduce the delay can be justified. Yet one more advantage of the PPM scheme is its inherently low electronic interference to other channels in application scenarios where an array of high-speed DMLs is required, since the electromagnetic radiation generated from the signal feeding lines can be cancelled at a distance away, which brings us the potential benefit in reducing the electronic crosstalk among multiple laser chips co-packaged inside a single module.

The rest of the paper is organized as follows: In Section 2, the dependence of the modulation response of the DPPM DFB on laser parameters, including the grating coupling coefficient, the cavity length, the facet reflectivity, the linewidth enhancement factor and the delay time is studied through numerical simulations; Section 3 shows the simulated device performance with an optimized DPPM DFB structure; and lastly, this work is summarized in Section 4.

## 2. Parameter Dependence of the Modulation Response

In the DPPM scheme, the CPR response restarts, and an effective detuning is introduced to the PPR. The detuning damps the resonance; hence, the PPR peak can be broadened. While the appearance of the PPR at the higher frequency (as compared to the CPR frequency) helps to raise the modulation bandwidth, the broadened PPR peak and the restarted CPR response mitigate the valley on the lower frequency side of the PPR peak. To obtain a broad and smooth modulation response, however, a quantitative study of the PPR position and broadness dependence on the DFB laser cavity parameters is still required in order to achieve the highest possible modulation bandwidth.

### 2.1. Simulation Model and Validation

**Figure 1:**
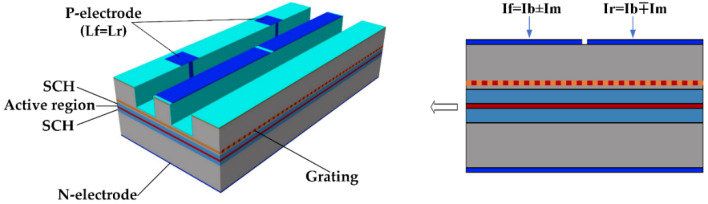
Schematic structure of the dual-sectional PPM-DFB laser.

The schematic structure of a dual-sectional PPM-DFB laser is shown in Figure 1. It is similar to the conventional single-sectional DFB laser except that the top electrode is divided into two electrically insulated parts, and the modulation current is a signal in differential mode. As a pulse is switched on or off, the rapid asymmetric change in the carrier density in each half of the device causes the PPR [34,38]. During the simulation of the dual-sectional DFB laser, we set a 10 μm insulated area (in actual processing, this is a groove etched into the lower layer of the cladding) between the two electrodes to prevent crosstalk. Since this work concerns the DFB laser cavity design only, a one-dimensional traveling wave model (1D TWM) suffices [42]:

(1) $$\frac{dN\left(z,t\right)}{dt}=\frac{\eta_{in}I\left(t\right)}{eV}-\frac{N\left(z,t\right)}{\tau_{c}}-\frac{v_{g}P_{s}\left(z,t\right)g\left(z,t\right)}{1+\varepsilon P_{s}\left(z,t\right)}$$

(2) $$\left(\frac{1}{v_{g}}\frac{\partial }{\partial t}+\frac{\partial }{\partial z}\right)F\left(z,t\right)=\left\{-j\delta +\frac{1}{2}\left[\frac{\Gamma g\left(z,t\right)}{1+\varepsilon P_{s}\left(z,t\right)}-\alpha \right]\right\}\cdot F\left(z,t\right)+j\kappa R\left(z,t\right)+{\tilde{s}}^{f}\left(z,t\right)$$

(3) $$\left(\frac{1}{v_{g}}\frac{\partial }{\partial t}-\frac{\partial }{\partial z}\right)R\left(z,t\right)=\left\{-j\delta +\frac{1}{2}\left[\frac{\Gamma g\left(z,t\right)}{1+\varepsilon P_{s}\left(z,t\right)}-\alpha \right]\right\}\cdot R\left(z,t\right)+j\kappa F\left(z,t\right)+{\tilde{s}}^{r}\left(z,t\right)$$

where N(z,t) is the carrier density, $\eta_{in}$ is the current injection efficiency, I(t) is the injected current, e is the electron charge, V is the active region volume, and $\tau_{c}$ is the carrier lifetime. $v_{g}=c/n_{g}$ is the group velocity, c is the speed of light in a vacuum, and $n_{g}$ is the group index. $g\left(z,t\right)=a\ln\left[N\left(z,t\right)/N_{0}\right]$ is the material optical gain, a is the material gain coefficient, and $N_{0}$ is the transparent carrier density. ε is the nonlinear gain suppression coefficient, F(z,t) is the slowly varying envelopes of the forward propagating fields, R(z,t) is the slowly varying envelopes of the backward propagating fields, j is the imaginary unit, Γ is the optical confinement factor, α is the optical modal loss, and κ is the grating coupling coefficient.

The photon density distribution is:

(4) $$P_{s}\left(z,t\right)=\frac{n_{eff}}{2h\nu_{0}}\sqrt{\frac{\varepsilon_{0}}{\mu_{0}}}\frac{\Gamma }{dwv_{g}}\cdot \left[{\left|F\left(z,t\right)\right|}^{2}+{\left|R\left(z,t\right)\right|}^{2}\right]$$

where $n_{eff}=n_{eff}^{0}-\lambda_{0}/4\pi \alpha_{LEF}\Gamma g\left(z,t\right)$ is the effective index, $n_{eff}^{0}$ is the effective index without injection, $\lambda_{0}$ is the peak gain wavelength, and $\alpha_{LEF}$ is the linewidth enhancement factor. h is the Planck’s constant, $\upsilon_{0}$ is the optical frequency corresponding to $\lambda_{0}$, $\varepsilon_{0}$ is the permittivity of a vacuum, $\mu_{0}$ is the permeability of a vacuum, d is the thickness of the active region, and w is the width of the active region.

The phase detuning factor from the Bragg wavelength is:

(5) $$\delta =\frac{2\pi }{\lambda_{0}}n_{eff}^{0}-\frac{1}{2}\alpha_{LEF}\Gamma g\left(z,t\right)-\frac{\pi }{\Lambda }$$

where Λ is the Bragg grating period.

The magnitude of the spontaneous emission noise fields ${\tilde{s}}^{f}\left(z,t\right)$ and ${\tilde{s}}^{r}\left(z,t\right)$ are approximated as Gaussian random processes with a zero mean and satisfy the following autocorrelation function [43]:

(6) $$\langle \left|{\tilde{s}}^{f,r}\left(z,t\right)\right|\left|{\tilde{s}}^{f,r}\left(z,t\right)\right|\rangle =2\sqrt{\frac{\mu_{0}}{\varepsilon_{0}}}\frac{\Gamma \gamma g_{sp}h\nu_{0}}{n_{eff}}\delta \left(z-z^{\prime }\right)\delta \left(t-t^{\prime }\right)$$

where γ indicates the spontaneous coupling factor, $g_{sp}$ indicates the spontaneous emission gain, and δ indicates Dirac’s delta function. The phase of the spontaneous emission noise fields is assumed to be uniformly distributed between 0~2π.

The finite bandwidth of the gain profile is modeled with an infinite impulse response (IIR) filter approach [44,45]:

(7) $${\left|H\left(\omega \right)\right|}^{2}=\left\{{\left(1-\eta \right)}^{2}/\left[1+\eta^{2}-2\eta \cos\left(\omega \Delta t\right)\right]\right\}$$

where η indicates the filter coefficient that controls the filter bandwidth, and Δt indicates the time marching step in simulation.

**Figure 2:**
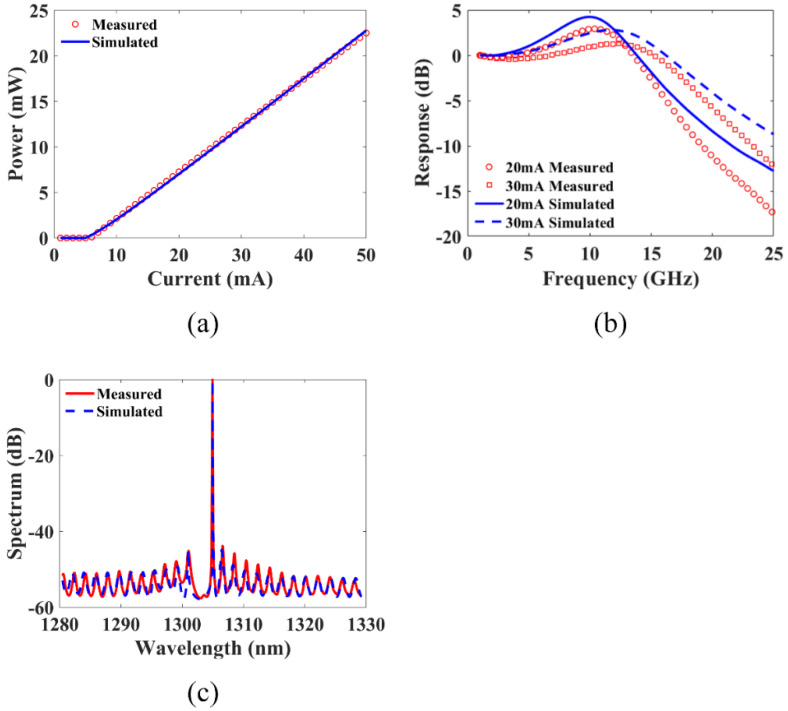
Comparison between measured and simulated results: (**a**) P–I curve; (**b**) Modulation response; (**c**) Spectrum.

Other than those cavity design parameters to be varied for performance optimization, the remaining DFB laser parameters are extracted by minimizing the error between the numerically calculated and the experimentally measured results. For a fabricated single-sectional DFB laser with its parameters given in the first 4 rows of Table 1, the remaining parameters in the table are obtained by searching for the best match of the calculated power–current (P–I) curve, small-signal intensity modulation response and spectrum to its measured counterparts, as shown in Figure 2.

The good agreement between the measured and simulated results verifies the consistency of our in-house simulation tool and the accuracy of the parameters. In the following dual-sectional DFB-laser simulation analysis, the cavity parameters, including the grating coupling coefficient, the cavity length and the facet reflectivities, will be varied within a reasonable range, whereas the remaining parameters will be fixed. Additionally, to streamline the analysis, the randomness of the front and back facet phases is ignored, and both phases are set to 0 in the following simulations. The effect of facet phases on the modulation response will be discussed once the structure is optimized.

### 2.2. Parameter Dependence of the PPR Frequency

**Figure 3:**
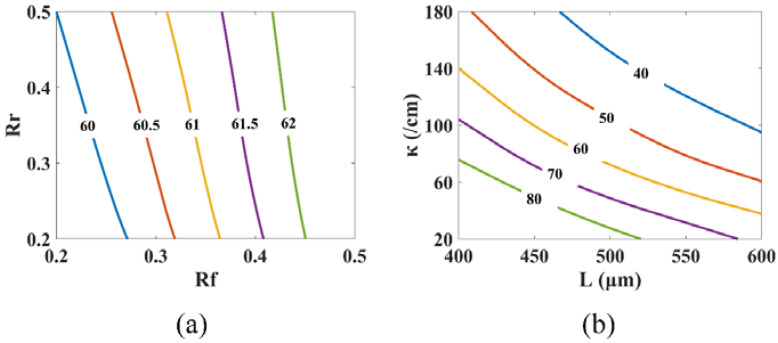
The PPR frequency dependence on parameters with $P_{bias}=12\;mW$: (**a**) PPR frequency vs. $R_{f}$ and $R_{r}$ with L=450 μm and κ=100/cm; (**b**) PPR frequency vs. *L* and κ with $R_{f}=R_{r}=0.2$.

**Figure 4:**
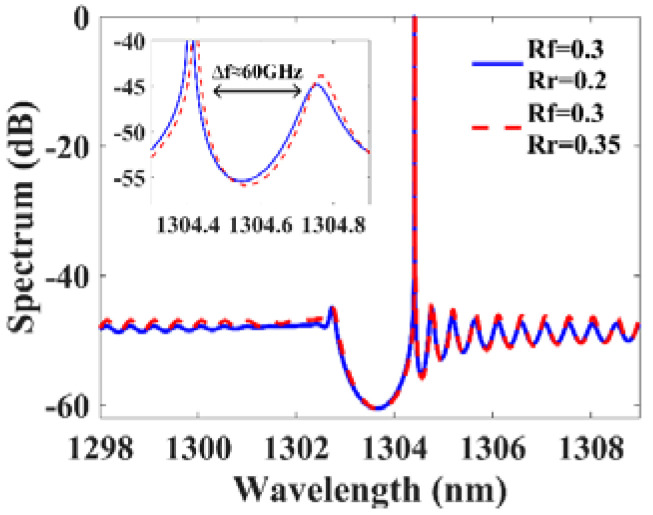
The lasing spectrum with L=450 μm and κ=100/cm.

Figure 3a shows the impact of facet reflectivity on the PPR frequency. With facet reflectivities ranging from 0.2 to 0.5, the PPR frequency can shift from 60 GHz to 62 GHz. As shown in the figure, the facet reflectivity has little effect on the PPR frequency. This is because the PPR frequency is determined by the spacing between the lasing mode and the closest Fabry–Perot (FP) mode, as shown in Figure 4, in agreement with the conclusion in [46]. The facet reflectivity has little impact on the spacing.

The effect of the grating coupling coefficient and cavity length on the PPR frequency is given in Figure 3b. The PPR frequency changes from 40 GHz to 80 GHz in a combined varying range of the cavity length from 400 μm to 600 μm and the grating coupling coefficient from 20/cm to 180/cm. With an increase in the grating coupling coefficient, the PPR frequency drops. The reason for this is that, as the grating coupling coefficient rises, the lasing spectrum’s stopband widens, resulting in a closer spacing between the lasing mode and the closest FP mode. As the cavity length rises, the PPR frequency also decreases. This is due to the fact that the spacing between the FP modes is inversely related to the laser cavity length. The spacing shrinks as the cavity length rises, resulting in a decreased frequency gap between the lasing mode and its neighboring FP mode. A general tendency is that the PPR frequency drops as the photons in the different modes take a longer time to travel around the laser cavity, since traveling through the cavity is a necessary condition for the photons in the different modes to establish a stable beating. Although an increase in either cavity length or coupling coefficient will reduce the PPR frequency, an increase in the cavity length is more efficient according to Figure 3b. For example, for a cavity length of 500 μm, quadrupling the coupling coefficient from 25/cm to 100/cm would drop the PPR frequency from 80 GHz to 50 GHz. For a coupling coefficient of 80/cm, dropping the PPR frequency from 80 GHz to 50 GHz, however, only needs an increase in the cavity length from 390 μm to 550 μm, approximately 1.4 fold. Therefore, for a given upper limit of the normalized coupling coefficient, we would pick a longer cavity length combined with a smaller coupling coefficient for an efficient reduction in the PPR frequency.

### 2.3. Parameter Dependence of the PPR Shape

As previously mentioned, in order to reduce the dip on the lower frequency side of the PPR peak and to prevent the bandwidth cut off, the PPR frequency needs to be reduced to some extent. On the other hand, if the PPR peak can be broadened, the expanded PPR peak tail can fill up the indentation, which also helps to raise the modulation bandwidth. To quantitively address the shape of the PPR peak, we define a factor as $S_{\mathrm{PPR}}{=H}_{\mathrm{PPR}}{\times F}_{\mathrm{PPR}}{/w}_{3\mathrm{dB}}$, where $F_{\mathrm{PPR}}$ is the PPR frequency, and $H_{\mathrm{PPR}}$ and $w_{3\mathrm{dB}}$ stand for the height and the full width at half maximum (FWHM) of the PPR peak, respectively.

**Figure 5:**
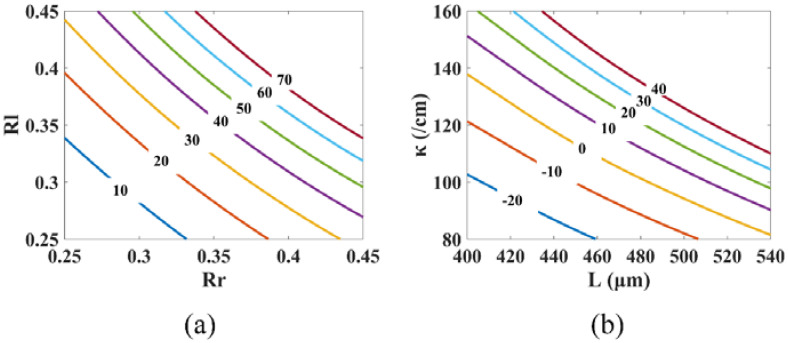
The PPR shape factor dependence on parameters with $P_{bias}=12\;mW$: (**a**) PPR shape factor vs. $R_{f}$ and $R_{r}$ with L=450 μm and κ=100/cm; (**b**) PPR shape factor vs. *L* and κ with $R_{f}=R_{r}=0.2$.

The effect of the facet reflectivity on the PPR shape factor is depicted in Figure 5a. With facet reflectivity ranging from 0.25 to 0.45, the PPR shape factor can change from 10 dB to 70 dB. As observed in the figure, the shape factor rises as the facet reflectivity rises. This is due to the fact that, as the facet reflectivity increases, the FP modes become more pronounced, which increases the intensity of the FP modes and, consequently, enhances the coupling between the lasing mode and its neighboring FP mode, sharpening the PPR peak. The front and back facet reflectivity have almost the same effect on the PPR shape factor.

Figure 5b shows that the PPR shape factor changes from −20 dB to 40 dB in a combined varying range of the cavity length from 400 μm to 540 μm and the grating coupling coefficient from 80/cm to 160/cm. As the cavity length and grating coupling coefficient rise, the PPR shape factor rises. This is because the increase in cavity length and grating coupling coefficient affects the single-mode stability of the laser. This instability enhances the coupling strength of the lasing mode and the neighboring FP modes, resulting in a sharp PPR peak. The effect of the cavity length on the shape factor is greater than that of the grating coupling coefficient.

### 2.4. Parameter Dependence of the Indentation Depth

The indentation depth is determined by the frequency of the PPR, the shape of the PPR and the decay of the intensity modulation response on the lower frequency side irrelevant to the PPR peak. The PPR frequency and shape are studied in the above sections. The decay of the response on the lower frequency side is dominated by the linewidth enhancement factor [38]. By adjusting the detuning between the peak gain wavelength and the Bragg wavelength, the linewidth enhancement factor usually varies between one and three.

**Figure 6:**
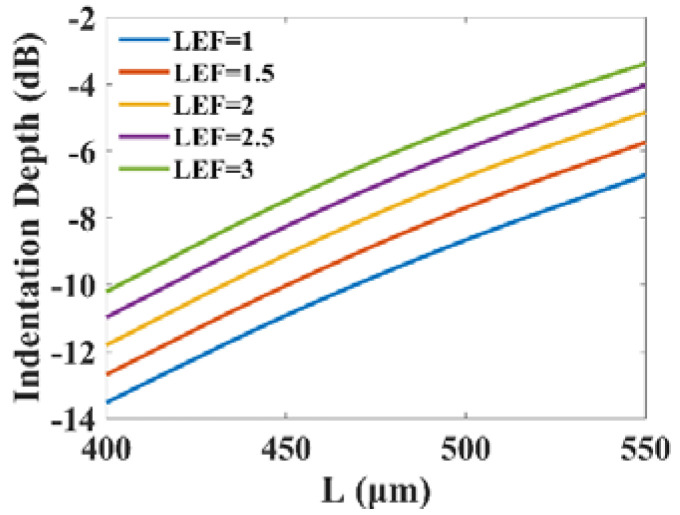
The indentation depth dependence on $\alpha_{LEF}$ for a few *L* with $P_{bias}=12\;mW$, κ=100/cm and $R_{f}=R_{r}=0.2$.

Figure 6 shows the effect of the linewidth enhancement factor on the indentation depth. The indentation depth can change from −13.7 dB to −3.5 dB in a combined varying range of the cavity length from 400 μm to 550 μm and the linewidth enhancement factor from one to three. It can be observed that, for different cavity lengths, the indentation becomes shallower as the linewidth enhancement factor increases. However, the indentation depth remains below −3 dB, and the bandwidth is still cut off by the rapid drop of intensity modulation response in the low frequency band.

### 2.5. The Effect of Time Delay

In the above sections, we learned that the PPR frequency, the PPR shape factor and the indentation depth can be varied by adjusting the laser cavity design parameters. However, the best effort is still not sufficient to eliminate the indentation on the lower frequency side of the PPR peak. In this section, we propose the DPPM scheme by introducing a time delay between the differential modes of the signal and study the impact of the delay time on the modulation response. In the experiments, the delay time can be achieved through two methods: using two transmission lines of different lengths to inject current or fabricating a delayed transmission line directly onto the subcarrier. The laser cavity design parameters are chosen in such a way that it gives the best possible intensity modulation response according to the above simulations when there is no time delay introduced. They are summarized in Table 2.

The injection currents at the front and rear electrodes are  $I_{f}=I_{b}+I_{m}\cos\left(\omega t\right)$ and $I_{r}=I_{b}-I_{m}\cos\left[\omega \left(t-\tau_{d}\right)\right]$ for small-signal analysis in DPPM, where $I_{b}$ is the DC bias current, $I_{m}$ is the small signal modulation current, $\tau_{d}$ is the delay time, and ω is the modulation frequency.

**Figure 7:**
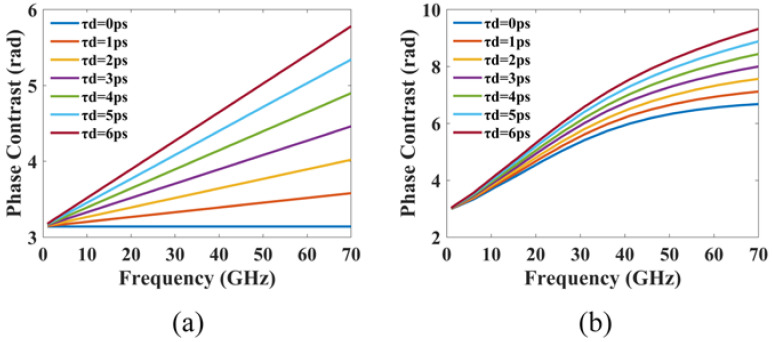
The effect of delay time $\tau_{d}$: (**a**) Phase contrast between injection currents vs. $\tau_{d}$; (**b**) Phase contrast between modulation responses vs. $\tau_{d}$.

As demonstrated in Figure 7a, the phase contrast between the two currents injected into the front and rear electrodes varies with modulation frequency except for the case where delay time is 0. The phase contrast between the modulation responses of injection currents $I_{f}=I_{b}+I_{m}\cos\left(\omega t\right),I_{r}=I_{b}$ and $I_{f}=I_{b},I_{r}=I_{b}-I_{m}\cos\left[\omega \left(t-\tau_{d}\right)\right]$ at different modulation frequencies is then calculated, and the results are presented in Figure 7b. The phase contrast between injected currents varies with the delay time, which leads to a variation in the phase contrast between modulation responses over delay time.

**Figure 8:**
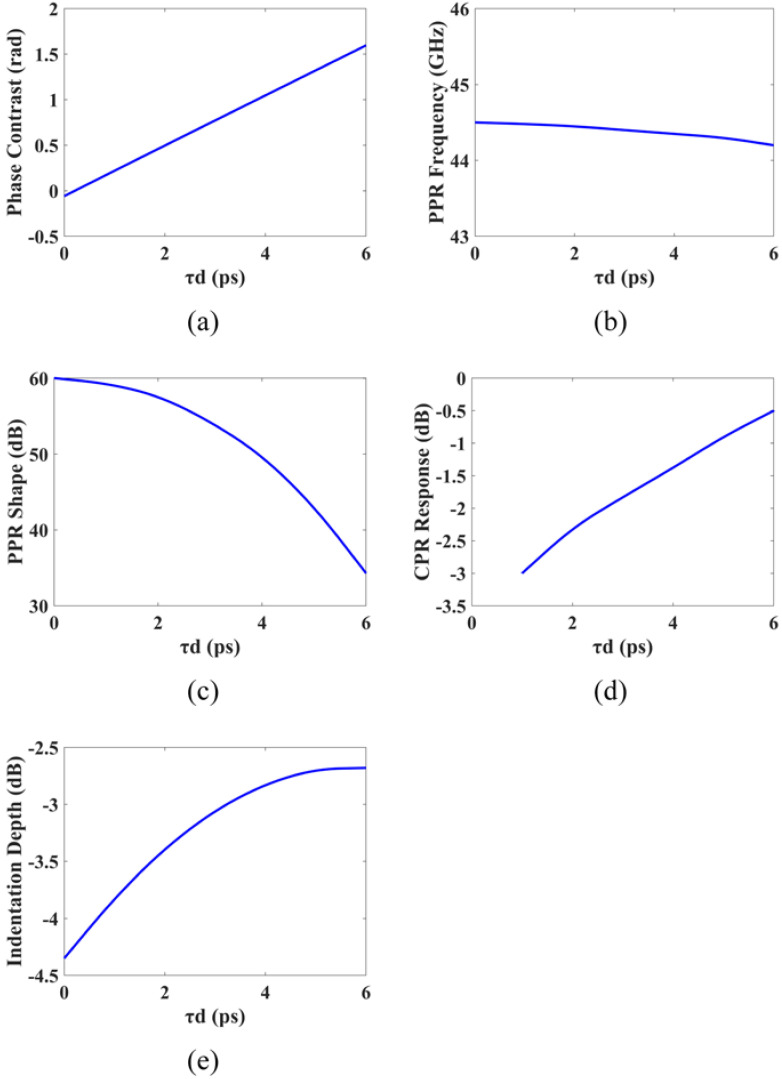
The effect of delay time $\tau_{d}$: (**a**) Phase contrast between modulation responses vs. $\tau_{d}$; (**b**) PPR frequency vs. $\tau_{d}$; (**c**) PPR shape factor vs. $\tau_{d}$; (**d**) CPR response vs. $\tau_{d}$; (**e**) Indentation depth vs. $\tau_{d}$.

Figure 8a shows the phase contrast between modulation responses when the modulation frequency is set to the PPR frequency. It is evident that the phase contrast is almost 0 when the delay time is 0. This results in the two modulation responses having the same frequency and phase, thereby generating the strongest resonance and leading to a sharp PPR peak. As the delay time increases, the phase contrast at the PPR frequency gradually increases, which causes the PPR detuning.

Figure 8b depicts the impact of the delay time on the average PPR frequency, which reveals that the delay time has little effect on the average PPR frequency. This is because introducing a delay time has almost no effect on the spacing between the lasing mode and the adjacent FP mode.

The effect of delay time on the PPR shape is shown in Figure 8c, from which it can be observed that the PPR shape factor decreases as the delay time increases. The longer the delay time is, the smaller the PPR shape factor will be, which can be attributed to the effective broadening of the PPR peak width by the large detuning of the PPR.

The relationship between delay time and CPR response is depicted in Figure 8d. It is worth noting that the conventional PPM method uses a pair of differential modulation signals as input, thereby maintaining a constant average carrier density and average photon density inside the cavity. Consequently, this results in the disappearance of the conventional CPR response. The DPPM scheme, on the other hand, introduces a time delay between the differential modulated currents, which alters the average carrier and photon densities within the cavity during the delay time and, thus, restarts the CPR response. As the delay time increases, the CPR response becomes more pronounced.

As evidenced in Figure 8e, by restarting the CPR response at the low-frequency region and broadening the PPR peak, these two effects work together to fill up the indentation on the lower frequency side of the PPR peak in the modulation response.

## 3. Optimized Cavity Structure and Simulated Performance

Table 3 lists the final optimized parameters based on 1D TWM. Using these parameters, we simulate the performance of the DPPM-DFB laser and compare it to that of the PPM-DFB laser and the conventional DML, all of which have the same structure except for the cavity length between the DPPM and PPM DFB and DML.

**Figure 9:**
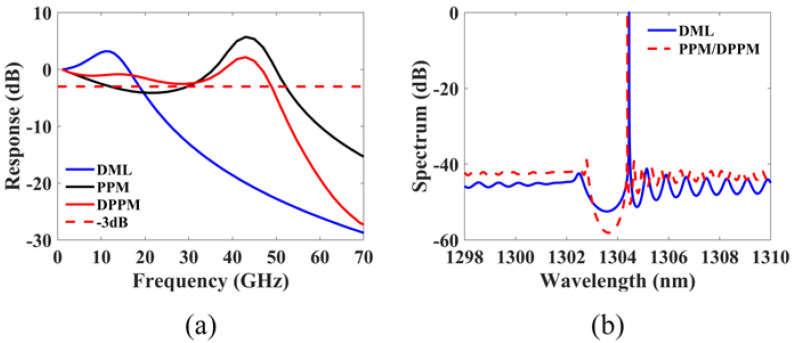
Simulation results: (**a**) Modulation response with $P_{bias}=12\;mW$; (**b**) Lasing spectrum.

Figure 9a shows the small-signal intensity modulation responses of the DPPM-DFB laser, the PPM-DFB laser and the conventional DML at a constant output power of 12 mW. The bias currents $I_{b}$ for the single-sectional and dual-sectional DFB lasers are 50 mA and 84.5 mA, respectively. As can be observed, the −3 dB bandwidth of the conventional DML is approximately 18.5 GHz. The PPM-DFB laser has a significant dip on the lower frequency side of the PPR peak, resulting in a bandwidth cut off at 11.5 GHz. The indentation on the modulation response is filled up in the DPPM-DFB laser, which allows its modulation bandwidth to be expanded to 49 GHz. The bandwidth of the DPPM-DFB laser is more than doubled compared with that of the conventional DML. The Lasing spectrums are shown in Figure 9b.

**Figure 10:**
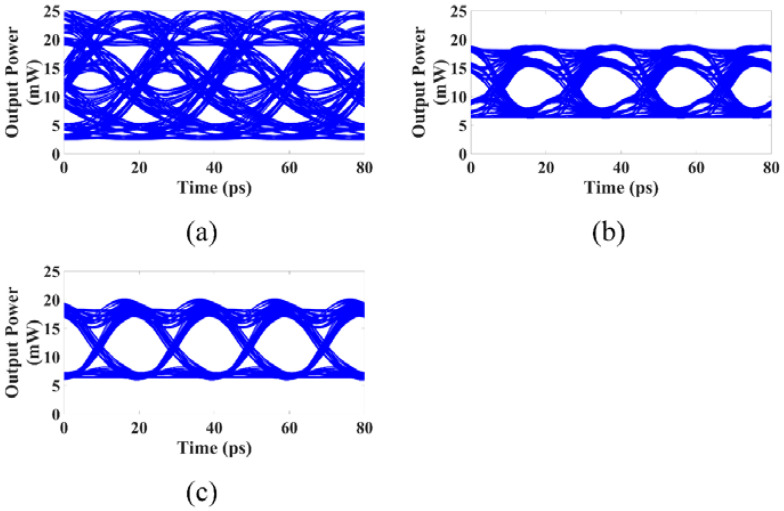
The 50 Gbps NRZ eye diagram with $P_{bias}=12\;mW$: (**a**) Conventional DML; (**b**) PPM-DFB laser; (**c**) DPPM-DFB laser.

**Figure 11:**
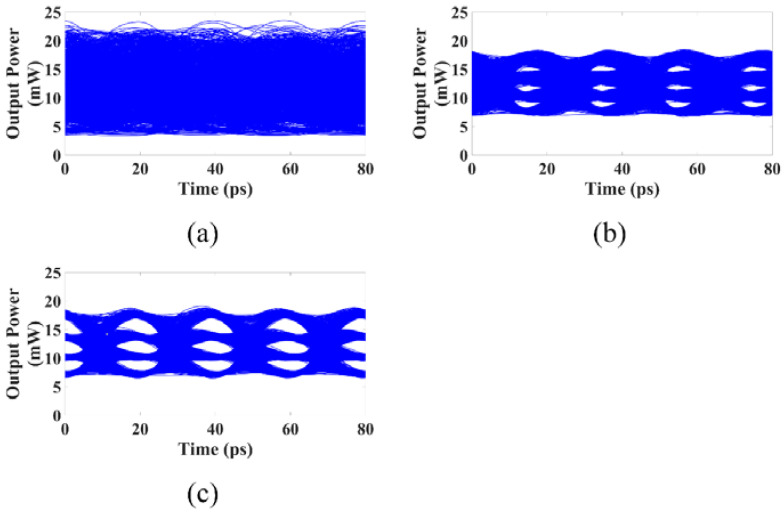
The 100 Gbps PAM4 eye diagram with $P_{bias}=12\;mW$: (**a**) Conventional DML; (**b**) PPM-DFB laser; (**c**) DPPM-DFB laser.

The large signal response of the above three different DFB lasers is simulated using NRZ signals and PAM4 signals, which are given in Figure 9 and Figure 10. The peak-to-peak modulation current of the NRZ signals is 0.5 $I_{b}$, and the peak-to-peak modulation currents of the PAM4 signals are 0.15 $I_{b}$ and 0.45 $I_{b}$. When using the NRZ signal with a modulation rate of 50 Gbps, the eye diagram obtained with the DPPM-DFB laser has the best quality, the eye diagram obtained with the conventional DML has a very poor quality and is almost closed, and the eye diagram quality obtained with the PPM-DFB laser is between the two, as shown in Figure 10. When employing the 100 Gbps PAM4 signal, the DPPM-DFB laser can still produce a good quality eye diagram, the conventional DML’s eye diagram is entirely closed, and the quality of the eye diagram obtained with the PPM-DFB laser is between the two, as shown in Figure 11. The modulation response of the PPM-DFB laser is substantially larger than that of the conventional DML when the modulation frequency is higher than 20 GHz despite the PPM-DFB laser having a narrower bandwidth than the conventional DML. The high-frequency response is important during high-speed modulation, which makes the PPM-DFB lasers’ large signal eye diagrams of higher quality than those of the conventional DML.

**Figure 12:**
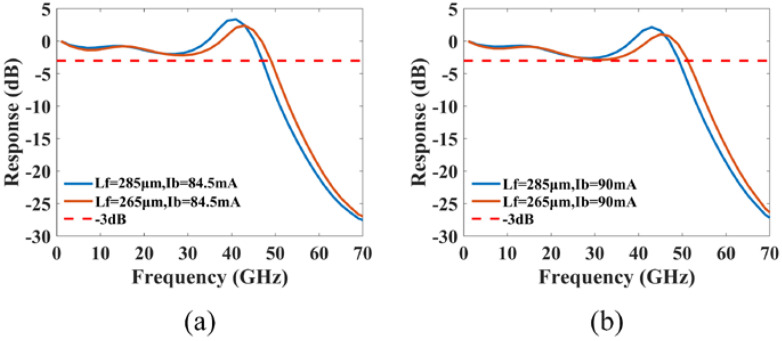
The effect of the errors between the front and rear cavity lengths and the design value on the modulation response: (**a**) $L_{r}=285\;\mu m$; (**b**) $L_{r}=265\;\mu m$.

**Figure 13:**
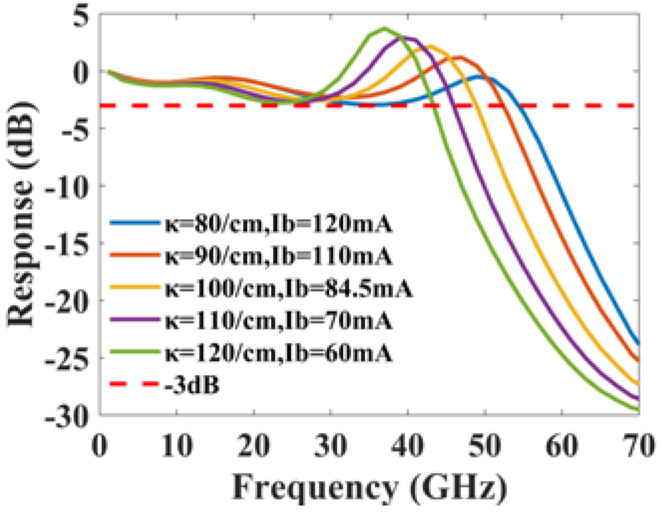
The effect of the errors between the grating coupling coefficient and the design value on the modulation response.

**Figure 14:**
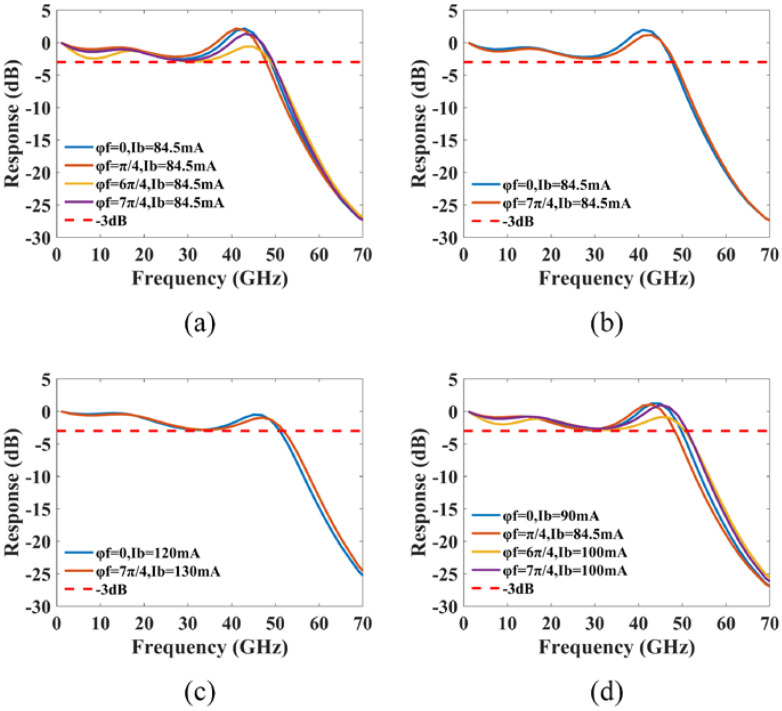
The effect of the front and back facet phases on the modulation response: (**a**) $\varphi_{r}=0$; (**b**) $\varphi_{r}=\pi /4$; (**c**) $\varphi_{r}=6\pi /4$; (**d**) $\varphi_{r}=7\pi /4$.

Considering the issues of structure errors and random facet phases during actual fabrication, the modulation response is then investigated in relation to the errors between the front and rear cavity lengths ($L_{f}$ and $L_{r}$) and the design value of 275 μm, the errors between the grating coupling coefficient and the design value of 100/cm, as well as the front and back facet phases ($\varphi_{f}$ and $\varphi_{r}$). As shown in Figure 12, when the errors between the front and rear cavity lengths and the design value are set to ±10 μm, all four cases can consistently reach a −3 dB bandwidth of nearly 50 GHz. Given that the dissociation error is typically only 5 μm (±2.5 μm), which is smaller than the simulation error we set, its impact on the modulation response can be ignored. The error in the grating coupling coefficient is varied within the range of 40/cm (±20/cm), and the results are depicted in Figure 13. We observe that, although the PPR frequency varies with the grating coupling coefficient, the delay time of 6 ps still effectively compensates the modulation response on the lower frequency side of the PPR peak. In order to analyze the effect of the front and back facet phases on the modulation response, we divided the front and back facet phases into eight equal parts in the range of 0~2π. Figure 14 shows that, out of the 64 phase combinations, 12 can achieve single-mode lasing with a −3 dB bandwidth of approximately 50 GHz by adjusting the bias current properly. The facet phases can affect the mode-spacing, causing variations in PPR frequency under different facet phase conditions. When the lengths of the front and rear cavities are unequal or the phases of the two facets are dissimilar, the front and rear cavities are not perfectly symmetrical, resulting in different modulation responses for each facet. This can be observed in Figure 12 and Figure 14, where the bias currents to obtain a 50 GHz bandwidth at the front and back facets may differ in cases of incomplete symmetry of the front and rear cavities.

## 4. Conclusions

In this work, the effects of the facet reflectivity, grating coupling coefficient, cavity length and linewidth enhancement factor on the PPR frequency, PPR shape and indentation depth are investigated through numerical simulation and analysis. A DFB laser with DPPM scheme is proposed by introducing a time delay between the two differential modes of the signal applied to the dual-sectional DFB to smooth out its intensity modulation response in the entire frequency range up to the PPR frequency. The PPR of the DPPM-DFB laser can be broadened and the CPR response will restart due to the detuning between the two signals in differential mode, which helps to fill up the dip on the lower frequency side of the PPR peak to avoid the cut-off of the modulation bandwidth at a much lower point than the PPR peak frequency. With a first order grating structure under the DPPM scheme, a modulation bandwidth close to 50 GHz is achievable with optimized cavity design parameters, which is more than double that in the conventional DML. Since the fabrication of the DPPM-DFB laser only requires mature technology without regrowth involved, it serves as a promising candidate as a light source for high-speed, fiber-optic communications systems where cost-effectiveness must be addressed.

## Figures and Tables

**Table 1:** Extracted parameters of the DFB laser.

Name	Symbol	Value	Unit
Bragg grating period	Λ	193.4	nm
Active region width	w	1.6	μm
Total quantum well thickness	d	0.04	μm
Cavity length	L	126	μm
Optical confinement factor	Γ	0.046	
Grating coupling coefficient	κ	167	$cm^{-1}$
Carrier lifetime	$\tau_{c}$	0.33	ns
Group index	$n_{g}$	3.6	
Material gain coefficient	a	1746.5	$cm^{-1}$
Transparent carrier density	$N_{0}$	$6.2\times 10^{17}$	$cm^{-3}$
Nonlinear gain suppression coefficient	ε	$6.2\times 10^{-17}$	$cm^{3}$
Optical modal loss	α	12	$cm^{-1}$
Amplitude reflectivity of front facet	$R_{f}$	0.1	
Phase of front facet	$\varphi_{f}$	1.72	rad
Amplitude reflectivity of back facet	$R_{r}$	0.91	
Phase of back facet	$\varphi_{r}$	3.09	rad
Effective index without injection	$n_{eff}^{0}$	3.37	
Linewidth enhancement factor	$\alpha_{LEF}$	2.1	
IIR filter coefficient	η	0.003	
Current injection efficiency	$\eta_{in}$	0.9	

**Table 2:** Cavity parameters of the DFB laser.

**Name**	**Symbol**	**Value**	**Unit**
Cavity length	L	550	μm
Grating coupling coefficient	κ	100	$cm^{-1}$
Amplitude reflectivity of front facet	$R_{f}$	0.25	
Amplitude reflectivity of back facet	$R_{r}$	0.25	

**Table 3:** Optimized parameters of the DFB laser.

Name	Symbol	Value	Unit
Bragg grating period	Λ	193.4	nm
Active region width	w	1.6	μm
Total quantum well thicknes	d	0.04	μm
Dual/Single-sectional DFB laser cavity length	L	550/275	μm
Optical confinement factor	Γ	0.046	
Grating coupling coefficient	κ	100	$cm^{-1}$
Carrier lifetime	$\tau_{c}$	0.33	ns
Group index	$n_{g}$	3.6	
Material gain coefficient	a	1746.5	$cm^{-1}$
Transparent carrier density	$N_{0}$	$6.2\times 10^{17}$	$cm^{-3}$
Nonlinear gain suppression coefficient	ε	$6.2\times 10^{-17}$	$cm^{3}$
Optical modal loss	α	12	$cm^{-1}$
Amplitude reflectivity of front facet	$R_{f}$	0.25	
Amplitude reflectivity of back facet	$R_{r}$	0.25	
Effective index without injection	$n_{eff}^{0}$	3.37	
Linewidth enhancement factor	$\alpha_{LEF}$	2.1	
IIR filter coefficient	η	0.003	
Current injection efficiency	$\eta_{in}$	0.9	
Differential delay time	$\tau_{d}$	6	ps

## Data Availability

Not applicable.
